# American Dental Society of Europe

**Published:** 1888-01

**Authors:** E. A. Galbreath

**Affiliations:** Hannover


### AMERICAN DENTAL SOCIETY OF EUROPE.

### FIFTEENTH ANNUAL MEETING AT COBLENTZ, GERMANY, SEPTEMBER, 1887.

REPORTED FOR THE INDEPENDENT PRACTITIONER BY DR. E. A. GALBREATH, HANNOVER.

CONTINUED FROM PAGE 659, VOL. VIII.

### THURSDAY AFTERNOON SESSION CONTINUED.

The President announced the Section of Dental Medicine.

*Dr. Elliot*—I have prepared no paper on the subject, because I know little about it, and it interests me very little. I made a few negative experiments with some new preparations, iodol, iodolized wax, cocaine, etc. I used for a short time a mixture of sulphuric acid ether and cocaine, advocated by Dr. Herbst, formulated by Dr. Taft, and made by Ash & Sons, but could derive no benefit from it. Ash & Sons have stopped making it. I have also used sublimate in solutions of one, two and three per cent., but with unsatisfactory results. Hydrogen peroxide I have also used a great deal, and with good results.

*Dr. Miller*—Our experience in the clinic has been that sublimate in all solutions invariably turns the tooth black in course of time. I do not find eugenol stronger than carbolic acid. The antiseptic power of iodoform comes from iodine evolved by a solution of the iodoform in fats, etc. The experiments showing that iodoform was not antiseptic were, therefore, most probably made under conditions where it was not soluble.

*Dr. Jenkins*—I would like to know if any one has noted the effects of cocaine in sub-cutaneous injections.

*Dr. Elliot*—Dr. Cunningham collected some statistics of cocaine used in this way, and finds the effects undesirable; it produces, not uncommonly, hysteria. The opinion of the profession in England is unfavorable to it.

*Dr. Field*—I use iodoform, with a two per cent. solution of bichloride of mercury, and have never seen a case of blackening of the tooth.

*Dr. Miller*—One-half per cent., or one to two hundred, is strong enough.

*Dr. Elliot*—I forgot to mention an invention of Dr. Rosenthal, for the treatment of dead teeth. He uses the exhaust from a current of water by an ejector. The tube cemented into the cavity, the air exhausted, water is let in and rushes into the vacuum, becomes discolored, and is let out. This process is repeated until the water comes out clean. It is not a great success practically, on account of the difficulty of connecting the tube air-tight into the tooth. The least movement of the apparatus loosens it. I made the same thing myself with an ordinary syringe.

The President announced the Section of Operative Dentistry.

*Dr. Kingsley*—Said that the greatest progress in Operative Dentistry has been in the direction of matrices, which contribute to the comfort of both the patient and the dentist. In looking back over twenty years, one finds that much has been done to make operations more complicated, but thanks to improved instruments, less fatiguing to the operator. But it is to be regretted that the poor patient at the other end of the instrument has not been a gainer in a proportionate degree. Recently, however, certain skillful operators have been bold enough to suggest that it is getting near the time when we should take more seriously into account the question of the wear and tear upon the nervous system of those who are so unfortunate as to need the application of our skill, and so fortunate as to be able to obtain it, and to question whether it is either necessary or justifiable to keep a patient confined three, four, six hours, as has been done, with the rubber-dam, gag, saliva pump, and numerous other blessings and curses combined, while the operator is pounding gold into a broken-down molar which loosens and drops out in another twelve months. The operator stops to eat a sandwich, and the poor victim of misguided and over zealous skill sucks a little brandy through a quill. This is no fancy picture. Such things have been done, boasted of by operators, and for years remembered and moaned over by the victims. It seems to us important for the dentist to take into consideration the life and health of the patient, as well as the small part of the patient's anatomy compressed in a single tooth. We regret that we cannot have Dr. Herbst with us at this meeting. We trust, however, that there are those present sufficiently conversant with his system to lead the discussion, and do justice to it—not making of it a question of country or of patriotism, as seemed to be the case before certain assemblies in America, judging from the reports in the dental journals. Science and art should know no country. With regard to implantation, it will be a source of regret if the searcher after hidden things duly elected by this society does not furnish us with a theory (we must have a theory) explaining how a tooth which has been out of the mouth a greater or less number of years, and has undergone all sorts of vicissitudes, can be planted in a hole artificially made in the human jaw, and there become solid.

*Dr. Bryan*—I have had one case of implantation where I extracted a badly abscessed first bicuspid. I waited several days until granulations set in, and then implanted, fastening the tooth to the adjoining bicuspid. It has been in since March 10th, and the last time I saw it, was doing nicely. The gum looked natural, and there was a hard, slightly swollen place over the tooth, apparently the development of bone.

*Dr. Elliot*—The root will be absorbed, and it will fall out.

*Dr. Miller*—The subject of implantation, as practiced by Dr. Younger, has perhaps more than any other occupied the minds of the profession, particularly in America, during the past year. You are all, no doubt, familiar with the operation. It consists in drilling or boring holes (sockets) in the alveolar process, at points where the natural teeth are missing, and planting or implanting teeth in these artificial alveoli. That teeth implanted in this manner will, as a rule, be retained for a certain length of time, there can be no doubt; how long, we are utterly unable to say. Dr. Younger uses for the purpose of implanting, not unfrequently, teeth which have been for days, months, and even years out of the mouth, and believes that the pericementum of such teeth becomes revitalized, so to speak, when they are planted in living tissue. In this, I think, there can be no doubt that Dr. Younger is entirely in error. When a tooth has been out of the mouth so long that the pericementum has become perfectly dry, there can be little hope of restoring it to life again. As to the manner in which such teeth are retained in the jaws for a certain time, I have already expressed myself in the INDEPENDENT PRACTITIONER for January, 1887. I have found by experiments on rabbits, that pieces of dead dentine may be retained and firmly held in living tissue by encapsulation. Small pieces brought under the skin or into the abdominal cavity under aseptic conditions, soon became enclosed and firmly held in a dense capsule of connective tissue, and could not be removed without tearing the tissue. In all cases, however, resorption soon began, forming irregular resorption territories or cavities, into which the tissue grew, thus forming another temporary bond of attachment between the dentine and the surrounding tissue. In one case, where the dentine was previously left for twenty-four hours in putrid saliva, suppuration took place and the piece was thrown off. When pieces of dentine having living pericementum were used, an apparently permanent union with the surrounding tissue was formed, no resorption being evident after three months. I see no physiological reason why the replantation of teeth, where the alveolar process has been sufficiently preserved to enable one to obtain a normally deep socket for the tooth, should not show nearly as large a percentage of successes as transplantation. The operation should always be performed under antiseptic conditions, and, so far as possible, freshly extracted teeth should be made use of.

*Dr. Elliot*—Will not absorption occur, even if the pericementum is healthy and good?

*Dr. Miller*—It may. The roots never exactly fit the bored holes, and the consequent irritation may cause them to be absorbed or thrown off. For that matter, even normal teeth will also be resorbed, I have seen roots with absorbed indentations, and the tooth held only by tissue that had grown into them.

*Dr. Jenkins*—I think in the majority of cases the success of this operation will depend upon the constitution of the patient. With some, liberties can be taken; with others, not. I once had a patient come to me complaining of some slight inconvenience from a pivot tooth, and I found the pivot ran through the side of the tooth into the alveolus at least half an inch. Another patient, away in the country, had a tooth extracted by the village barber, and the next day, dissatisfied with her appearance, she replaced it herself. In a few days she came to me in great pain, and with an abscess, which just then I had no time to treat, and, therefore, postponed for a few days. She did not come back for six months, when I treated the abscess and made a gold filling in the tooth. It seemed to be firm and in serviceable condition, and for all that I know it may remain so to this day.

*Dr. Field*—I have implanted many teeth, but the roots have all resorbed.

(TO BE CONTINUED.)

